# Incidence and factors associated with cutaneous immune-related adverse events to immune check point inhibitors: An ambispective cohort study

**DOI:** 10.3389/fimmu.2022.965550

**Published:** 2022-10-20

**Authors:** Athitaya Luangnara, Salin Kiratikanon, Thanika Ketpueak, Thatthan Suksombooncharoen, Chaiyut Charoentum, Busyamas Chewaskulyong, Napatra Tovanabutra, Siri Chiewchanvit, Surapon Nochaiwong, Mati Chuamanochan

**Affiliations:** ^1^ Maharaj Nakorn Chiang Mai Hospital, Chiang Mai, Thailand; ^2^ Division of Dermatology, Department of Internal Medicine, Chiang Mai University, Chiang Mai, Thailand; ^3^ Division of Oncology, Department of Internal Medicine, Chiang Mai University, Chiang Mai, Thailand; ^4^ Pharmacoepidemiology and Statistics Research Center, Faculty of Pharmacy, Chiang Mai University, Chiang Mai, Thailand; ^5^ Department of Pharmaceutical Care, Faculty of Pharmacy, Chiang Mai University, Chiang Mai, Thailand

**Keywords:** anti-programmed cell death-1, anti-programmed cell death ligand-1, cutaneous immune-related adverse event, cytotoxic T-lymphocyte antigen-4 inhibitors, immune checkpoint inhibitors

## Abstract

**Background:**

Although immune checkpoint inhibitors (ICIs) have become the frontline treatment option for patients with various advanced cancers due to improved survival, they can be associated with a spectrum of cutaneous immune-related adverse events (cirAEs). However, little is known regarding the occurrence and patterns of cirAE-related ICI therapy in patients of different races other than white populations. Therefore, we investigated the incidence and associated factors of cirAEs among cancer patients in northern Thailand.

**Methods:**

A referral-center-based ambispective cohort study was conducted from January 1, 2017, to March 31, 2021. Based on a linked database and merged patient-level data, adult patients with pathologically confirmed cancer who were diagnosed and received ICI therapy regardless of cancer type and followed up through August 31, 2021, were included. All cirAE-related ICI therapy was based on clinical evaluation and ascertainment by a board-certified dermatologist. The incidence of cirAE-related ICI therapy with confidence intervals (CIs) across cancer- and ICI therapy-specific groups was estimated. Factors associated with cirAEs were evaluated using multivariable modified Poisson regression to estimate risk ratios (RRs) and 95% CIs.

**Results:**

The study included 112 patients (67 men [59.8%]; mean age, 65.0 [range, 31.0-88.0] years), who were mainly diagnosed with lung cancer (56.3%), followed by liver cancer (19.6%). The overall incidence of cirAE-related ICI therapy was 32.1% (95% CI, 24.1-41.4); however, there was no substantial difference in sex, cancer type, or individual ICI therapy. The two identified prognostic risk factors of cirAE-related ICI therapy were age >75 years (adjusted RR, 2.13; 95% CI, 1.09-4.15; *P*=0.027) and pre-existing chronic kidney disease stages 3-4 (adjusted RR, 3.52; 95% CI, 2.33-5.31; *P*<0.001).

**Conclusions:**

The incidence of cirAE-related ICI therapy among Thai cancer patients was comparable to that in white populations. Early identification, particularly in elderly patients and those with CKD, should be implemented in clinical practice to help optimize therapeutic decision-making and patient health outcomes.

## Introduction

Over the past decade, immune checkpoint inhibitors (ICIs) have become the frontline treatment option and have revolutionized the management of various advanced cancers (1, 2). ICIs target immune system activation against cancer cells; specifically, these monoclonal antibodies are cell surface proteins (i.e., cytotoxic T-lymphocyte antigen-4 [CTLA-4]) and programmed cell death-1 (PD-1)/programmed cell death ligand-1 (PD-L1) (1). In practice, anti-CTLA-4 (e.g., ipilimumab), anti-PD-1 (e.g., nivolumab or pembrolizumab), and anti-PD-L1 (e.g., atezolizumab or durvalumab) agents have been widely used either as monotherapy or in combination for the treatment of advanced solid tumors (e.g., breast, lung, head and neck, renal and bladder, skin cancers, cervical cancer, colorectal cancer, and hepatocellular carcinoma) and hematologic malignancies (1–3).

Given that ICIs act *via* nonspecific activation of the immune system, treatment with ICIs can also induce immune-related toxicities that mimic autoimmune diseases. Theoretically, these immune-related adverse events can affect any organ system; however, the skin is one of the most common organs involved (4, 5). Clinical characteristics, skin manifestations, and severity of cutaneous immune-related adverse events (cirAEs) vary across ICI therapies and comorbid conditions (5, 6). Existing studies among the white population in several western countries have reported the incidence of cirAEs ranging from 13.1% to 52.3% and are more common in anti-CTLA-4 than in anti-PD-1/anti-PD-L1 (5, 7–12).

Despite the occurrence and patterns of cirAE-related ICI therapy that have been extensively reported, particularly in melanoma and lung cancer (5, 10–12), to our knowledge, limited evidence exists regarding the incidence and risk factors of cirAEs in different cancer types and non-white populations. To date, data on the epidemiology of cirAE-related ICI therapy among advanced cancer patients in Thailand are limited, with lung and liver cancers being the most common malignancies. To better understand and improve treatment care for patients receiving ICIs therapy, we aimed to investigate the incidence and risk factors of cirAEs among cancer patients in a large tertiary care referral center in northern Thailand.

## Methods

### Study design and patient population

This single-center, ambispective (retrospective and prospective) study was based on a cohort of cancer patients from the Maharaj Nakorn Chiang Mai Hospital, Chiang Mai University, a tertiary referral and university hospital in Northern Thailand. The study protocol was conducted according to the Declaration of Helsinki and reported following the Strengthening the Reporting of Observational Studies in Epidemiology (STROBE) statement (13) along with the Reporting of studies Conducted using Observational Routinely collected health Data (RECORD) statement and extension to STROBE reporting guidelines (14). All consecutive cancer patients who received ICIs for treatment of any cancer type between January 1, 2017, and March 31, 2021, and screened for inclusion with follow-up through August 31, 2021, were included in the study. For prospective data collection, data were collected after the approval of the institutional review board of the Faculty of Medicine, Chiang Mai University (study code: MED-2562-06587). The requirement for informed consent for the retrospective phase was waived because of the nature of the study, and written informed consent was obtained for the prospective phase. No financial compensation was provided for participating in this study, and the patient information was de-identified.

Relevant information was gathered and merged using the local joint databases from the Maharaj Nakorn Chiang Mai Hospital based on (i) routine cancer care management profiles, including patient-level details, which comprised clinical characteristics, cancer types, cancer stage, Eastern Cooperative Oncology Group (ECOG) performance status, pre-ICI therapy (traditional chemotherapy, radiotherapy, targeted agent, concurrent chemoradiation therapy, traditional chemotherapy plus radiotherapy, or none), comorbidities (hypertension, diabetes, coronary artery [CAD], chronic kidney disease [CKD] stage 3-4, chronic pulmonary disease, cirrhosis, Charlon comorbidity index, and multimorbidity condition), history of drug allergy, ICI therapy (anti-PD-1/anti-PD-L1 and or anti-CTLA-4 compared), and patient monitoring and treatment follow-up; and (ii) electronic health records, claim databases that contain outpatient and inpatient data, pharmacy dispensing, and laboratory results. The final datasets were reviewed and cross-checked to ensure data quality and to limit the quantity of missing data.

Patients eligible for this study included new users aged 18 years or older with pathologically confirmed cancer diagnosed and treated with at least one dose of ICI therapy (monotherapy or based on two-drug combinations) regardless of cancer type. Exclusion criteria were (i) a history of a cutaneous event within the first three months before receiving ICI therapy; (ii) incomplete data on ICI treatment and monitoring during the follow-up period; (iii) a history of end-stage kidney disease, serum aminotransferase/serum alanine aminotransferase more than 3-times the reference upper limit of normal, or total bilirubin more than 2-times the upper limit of normal; and (iv) pregnancy or breastfeeding.

### Outcome: cirAEs

We screened for possible cirAEs using a manual review of routine cancer care management profile information and medical records. All eligible cirAEs were based on clinical evaluation and judgment that established treatment-emergent reactions after ICI therapy based on cutaneous morphology, in which skin lesions exist for more than one day and involve a body surface area of at least 1% or more (10, 15). Details regarding the clinical consequences of cirAE-related ICI therapy were collected, including date of onset, the severity of symptoms using the Common Terminology Criteria for Adverse Events (CTCAE) version 5.0 (16), and clinical complications and associated hospitalization. Two or more study investigators, of which at least one is a board-certified dermatologist (MC), were critically reviewed, and all cirAE cases were ascertained in terms of causality related to ICI therapy.

### Statistical analysis

The sample size of the study was estimated based on the occurrence of cirAEs among cancer patients who received ICI therapy and ranged from 13.1% to 52.3% (5, 7–12). To account for a type I error of 0.05 and compensate for 10% missing data, at least 99 cancer patients receiving ICI therapy were required for this study. Descriptive data are summarized as numbers with percentages for categorical variables and mean ± standard deviation (SD) or medians with interquartile range (IQR) as appropriate. Differences in cirAE-related ICI therapy status (with or without cirAEs) were assessed using Fisher’s exact test and independent t-test or Wilcoxon rank-sum test for categorical and continuous data, respectively. The incidence estimated with 95% confidence intervals (CIs) of the occurrence of cirAE-related ICI therapy, as well as cirAE-specific morphology, were calculated based on age (<65, 65-75, and >75 years), sex (male and female), cancer types (lung, liver, melanoma, and others), pre-ICI therapy (traditional chemotherapy, radiotherapy, targeted agents, concurrent chemoradiation therapy, and traditional chemotherapy plus radiotherapy), and ICI therapy (anti-PD-1, anti-PD-L1, and anti-CTLA-4 or anti-CTLA-4 plus anti-PD-1/PD-L1).

To identify and explore the predictors associated with the development of cirAE-related ICI therapy, candidate risk factors based on all available patient characteristics, cancer-related characteristics, comorbidities, history of drug allergy, laboratory profiles, and phase of data collection (retrospective vs. prospective) were employed using univariable modified Poisson regression models with robust standard errors to estimate crude relative risks (RR) and 95% CIs. Next, candidate predictors with a *P*-value <0.100 were analyzed in the multivariable modified Poisson regression models using the stepwise backward method. The final model was determined for multicollinearity by investigating the variance inflation factors of the risk factors in the multivariable model. Multiple imputation analysis was performed to account for missing values; however, variables with more than 20% missing data were excluded from the analysis. All analyses were performed using Stata version 16.0 (Stata Corporation, College Station, TX, USA). Two-tailed tests with a *P*-value <0.05 were considered statistically significant for all tests.

## Results

### Characteristics of cancer patients treated with ICI therapy

A total of 118 cancer patients were screened. Of these, four patients were excluded according to the study selection criteria, resulting in 112 patients (64 patients in the retrospective cohort and 48 patients in the prospective cohort) being included in this study (**Supplementary Figure S1**). Overall, most patients were male (59.8%), with a mean age of 65.9 ± 10.3 years. The included patients were mainly diagnosed with lung cancer (56.3%), followed by liver cancer (19.6%). According to cancer stage, 72.1% of the patients had stage IV cancer, while only 15.2% had an ECOG performance of grade 0. Regarding cancer management, traditional chemotherapy (50.5%) and anti-PD-1 therapy (nivolumab and pembrolizumab, 56.2%) were the most commonly prescribed pre-ICI and ICI therapies, respectively. **Table 1** describes the details of patient characteristics according to cirAE-related ICI therapy status.

**Table 1:** Patient characteristics according to cirAE-related ICI therapy status.

Characteristic	Overall (n=112)	With cirAE (n=36)	Without cirAE (n=76)	*P* value
Age, mean (SD), year	65.0 (10.3)	66.8 (10.3)	64.2 (10.2)	0.202
<65	57 (50.9)	14 (38.9)	43 (56.6)	0.120
65-75	39 (34.8)	14 (38.9)	25 (32.9)	
>75	16 (14.3)	8 (22.2)	8 (10.5)	
Sex				
Male	67 (59.8)	22 (61.1)	45 (59.2)	1.000
Female	45 (40.2)	14 (38.9)	31 (40.8)	
Cancer type				
Lung	63 (56.3)	17 (47.2)	46 (60.5)	0.564
Liver	22 (19.6)	9 (25.0)	13 (17.1)	
Melanoma	11 (9.8)	4 (11.1)	7 (9.2)	
Other^†^	16 (14.3)	6 (16.7)	10 (13.2)	
Cancer stage^‡^				
II/III	31 (27.9)	11 (30.6)	20 (26.7)	0.659
IV	80 (72.1)	25 (69.4)	55 (73.3)	
ECOG performance scale^‡^				
0	17 (15.2)	4 (11.1)	13 (17.1)	0.069
1	82 (73.2)	31 (86.1)	51 (67.1)	
2	13 (11.6)	1 (2.8)	12 (15.8)	
Pre-ICI therapy^‡^				
Traditional chemotherapy	56 (50.5)	15 (41.7)	41 (54.7)	0.465
Radiotherapy	5 (4.5)	1 (2.8)	4 (5.3)	
Targeted agent	23 (20.7)	9 (25.0)	14 (18.7)	
Concurrent chemoradiation therapy	5 (4.5)	2 (5.6)	3 (4.0)	
Traditional chemotherapy plus radiotherapy	6 (5.4)	1 (2.8)	5 (6.7)	
None	16 (14.4)	8 (22.2)	8 (10.7)	
ICI precipitating first cirAE				
Anti-PD-1	63 (56.2)	21 (58.3)	42 (55.2)	0.833
Anti-PD-L1	44 (39.3)	13 (36.1)	31 (40.8)	
Anti-CTLA-4 or anti-CTLA-4 plus anti-PD-1/PD-L1	5 (4.5)	2 (5.6)	3 (4.0)	
Comorbid conditions				
Hypertension	39 (34.8)	16 (44.4)	23 (30.3)	0.202
Diabetes mellitus	18 (16.1)	9 (25.0)	9 (11.8)	0.099
Coronary artery disease	3 (2.7)	2 (5.6)	1 (1.3)	0.241
Chronic kidney disease	4 (3.6)	4 (11.1)	0 (0.0)	0.009
Chronic pulmonary disease	7 (6.2)	1 (2.8)	6 (7.9)	0.426
Cirrhosis	13 (11.6)	4 (11.1)	9 (11.8)	1.000
Charlson comorbidity index, median (IQR)	8 (7-9)	8 (8-9.5)	8 (7-9)	0.260
Multimorbidity (excluded cancer)	23 (20.5)	10 (27.8)	13 (17.1)	0.216
History of drug allergy	21 (18.8)	6 (16.7)	15 (19.7)	0.799
Laboratory variables				
Hemoglobin, g/dL	11.5 (1.9)	11.6 (2.0)	11.5 (1.9)	0.842
White blood cells count x 10^3^, µ/L, median (IQR)	6.8 (5.4-8.8)	7.0 (5.3-8.8)	6.7 (5.4-8.8)	0.728
Platelet count x 10^3^, µ/L, median (IQR)	255 (195-344)	235 (190-351)	264 (200-338)	0.691
Neutrophil-to-lymphocyte ratio, median (IQR)	2.7 (1.9-5.1)	2.2 (1.7-3.5)	3.0 (2.0-5.3)	0.069
Platelet-to-lymphocyte ratio, median (IQR)	162.8 (118.4-231.7)	154.8 (95.8-231.8)	169.9 (122.9-231.7)	0.320
Serum albumin, g/dL	3.8 (0.6)	3.8 (0.6)	3.8 (0.6)	0.799
Total protein, g/dL	7.6 (1.0)	7.7 (1.0)	7.5 (0.7)	0.158
Globulin, g/dL	3.7 (0.8)	3.8 (0.9)	3.6 (0.7)	0.191
Albumin-to-globulin ratio	1.1 (0.3)	1.1 (0.3)	1.1 (0.3)	0.546
Aspartate aminotransferase, U/L, median (IQR)	19 (14-32)	19 (13-51.5)	19 (14-27)	0.344
Alanine transaminase, U/L, median (IQR)	23 (19-36)	26 (18.5-46)	23 (19-31)	0.728
Alkaline phosphatase, U/L, median (IQR)	91 (71-122)	101.5 (71-120)	86 (70-130)	0.794
Data collection				
Prospective cohort	48 (42.9)	21 (58.3)	27 (35.5)	0.026
Retrospective cohort	64 (57.1)	15 (41.7)	49 (64.5)	

Values are numbers with percentages in parentheses or expressed as mean (SD), unless otherwise indicated.

^†^Other cancer types included head and neck, genitourinary (renal, urothelial), bladder cancer, colorectal carcinoma, cervical cancer, and gastric cancer.

^‡^Missing data for one patient.

cirAEs, cutaneous immune-related adverse events; CTLA-4, cytotoxic T-lymphocyte antigen-4; ECOG, Eastern Cooperative Oncology Group; ICI, immune checkpoint inhibitor; IQR, interquartile range; PD-1, programmed cell death-1; PD-L1, programmed cell death-1-ligand 1.

### Incidence of cirAEs among ICI recipients

The overall unadjusted incidence for cirAE-related ICI therapy was 32.1% (95% CI, 24.1-41.4; **Table 2**). According to ICI therapy, the unadjusted incidences of cirAE-related ICI therapy were 33.3% (95% CI, 22.7-45.9) for anti-PD-1, 29.5% (95% CI, 17.9-44.7) for anti-PD-L1, and 40.0% (95% CI, 9.8-80.3) for anti-CTLA-4 or anti-CTLA-4 plus anti-PD-1/PD-L1. Remarkably, a high incidence of cirAE-related ICI therapy was observed among individuals aged >75 years (50.0%; 95% CI, 27.1-72.9). However, there was no substantial difference in the sex and type of cancer-specific incidence of cirAE-related ICI therapy.

**Table 2:** Age-, sex-, and cancer-specific conditions incidence of cirAE in patients treated with ICIs.

Characteristics	N	Incidence of cirAE-related ICI therapy		
		Cases	Incidence Estimated (95% CI)
**Age, year**			
<65	57	14	24.6 (15.0 – 37.5)
65-75	39	14	35.9 (22.4 – 52.0)
>75	16	8	50.0 (27.1 – 72.9)
**Sex**			
Male	67	22	32.8 (22.6 – 45.0)
Female	45	14	31.1 (19.3 – 46.1)
**Cancer type**			
Lung	63	17	27.0 (17.4 – 39.3)
Liver	22	9	40.9 (22.7 – 62.0)
Melanoma	11	4	36.4 (14.2 – 66.4)
Other^†^	16	6	37.5 (17.8 – 62.5)
**Pre-ICI therapy** ^‡^			
Traditional chemotherapy	56	15	26.7 (16.7 – 40.0)
Radiotherapy	5	1	20.0 (26.5 – 69.6)
Targeted agent	23	9	39.1 (21.6 – 60.0)
Concurrent chemoradiation therapy	5	2	40.0 (9.8 – 80.3)
Traditional chemotherapy plus radiotherapy	6	1	16.7 (2.2 – 63.7)
None	16	8	50.0 (27.1 – 72.9)
**ICI therapy**			
Anti-PD-1	63	21	33.3 (22.7 – 45.9)
Anti-PD-L1	44	13	29.5 (17.9 – 44.7)
Anti-CTLA-4 or anti-CTLA-4 plus anti-PD-1/PD-L1	5	2	40.0 (9.8 – 80.3)
**Overall**	**112**	**36**	**32.1 (24.1 – 41.4)**

^†^Other cancer types included head and neck, genitourinary (renal, urothelial), bladder cancer, colorectal carcinoma, cervical cancer, and gastric cancer.

^‡^Missing data for one patient.

CI, confidence interval; cirAEs, cutaneous immune-related adverse events; CTLA-4, cytotoxic T-lymphocyte antigen-4; ICI, immune checkpoint inhibitor; PD-1, programmed cell death-1; PD-L1, programmed cell death-1-ligand 1.

**Figure 1:**
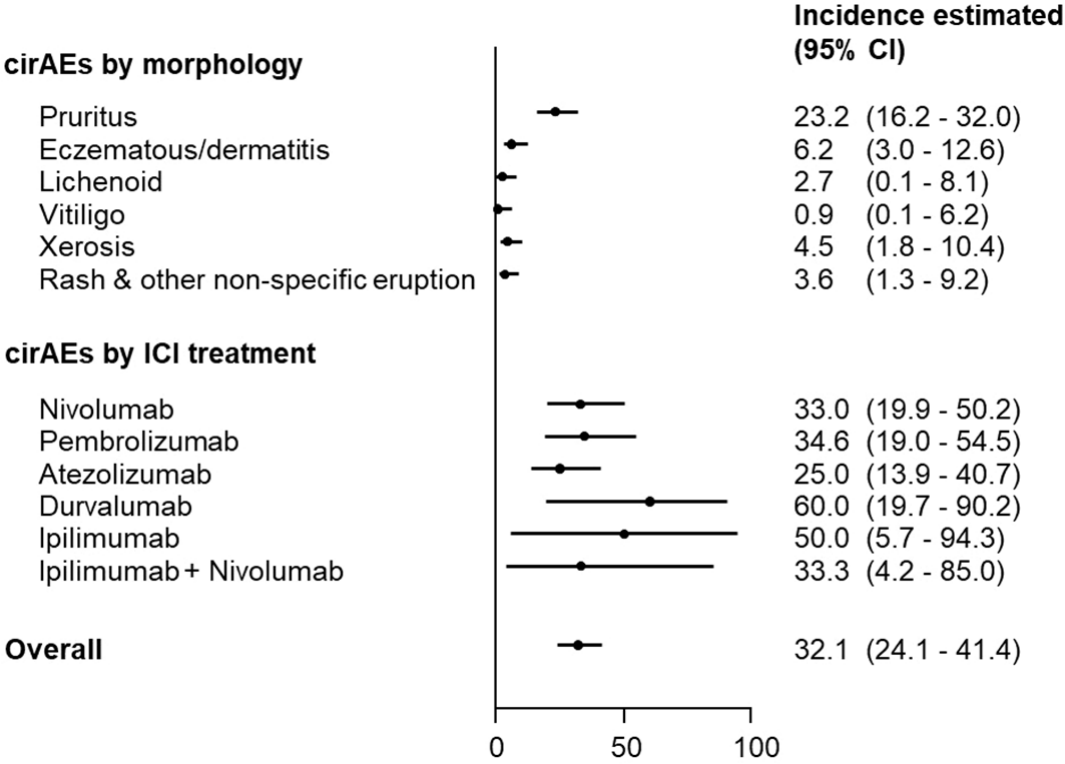
Incidence of cirAE-related ICI therapy according to morphology and individual ICI therapy. CI, confidence interval; cirAEs, cutaneous immune-related adverse events; ICI, immune checkpoint inhibitor.

No statistically significant differences were observed with respect to individual ICI therapies (**Figure 1**). Based on morphology-specific cirAEs (**Figure 1**), isolated pruritus was the most cirAE-related to ICI therapy (23.2%; 95% CI, 16.2-32.0), followed by eczematous/dermatitis (6.2%; 95%, 3.0-12.6), and skin xerosis (4.5%; 95% CI, 1.8-10.4). Moreover, rash and other non-specific eruption (3.6%; 95% CI, 1.3-9.2), lichenoid (2.7%; 95%, 0.1-8.1), and vitiligo (0.9%; 95% CI, 0.1-6.2) were also diagnosed as cirAEs among ICI recipients.

Among individuals with cirAE-related ICI therapy, initial adverse cutaneous reactions emerged with a median time of 67 days (IQR, 21-146 days) after ICI initiation. Specifically, the median time of cirAEs was 42 days (IQR, 15-84 days) for anti-PD-1; 150.5 days (91-371.5 days) for anti-PD-L1; and 87 days (IQR, 45-782 days) for anti-CTL4 or anti-CTLA-4 plus anti-PD-1/PD-L1 (*P*=0.941). Nevertheless, no patients who required hospitalization or emergency department visits due to cirAE-related symptoms were identified. Most of the cirAE-related ICI therapy was grade 1, based on CTCAE version 5.0.

### Risk factors of cirAEs among ICI recipients

Based on patient demographics, the univariable modified Poisson regression identified seven candidate predictors with P<0.100. These included age, pre-ICI therapy, diabetes, CAD, CKD stage 3-4, neutrophil-to-lymphocyte ratio, and data collection (**Supplementary Table S1**). Subsequently, two independent significant risk factors of cirAE-related ICI therapy were identified based on the multivariable modified Poisson regression (**Table 3**): (i) older age over 75 years (adjusted RR, 2.13; 95% CI, 1.09-4.15; *P*=0.027) and (ii) history of CKD stage 3-4 (adjusted RR, 3.52; 95% CI, 2.33-5.31; *P*<0.001).

**Table 3:** Multivariable risk factors of cirAE in patients treated with ICIs (n=112).

Factors	Adjusted RR (95% CI)	*P* Value
**Age, year**		
<65	1.00 (Reference)	
65-75	1.28 (0.69 – 2.38)	0.439
>75	2.13 (1.09 – 4.15)	0.027
**Chronic kidney disease: stage 3-4**		
No	1.00 (Reference)	
Yes	3.52 (2.33 – 5.31)	<0.001

CI, confidence interval; cirAEs, cutaneous immune-related adverse events; ICI, immune checkpoint inhibitor; RR, risk ratio.

## Discussion

This study investigated the occurrence of cirAEs in adult cancer patients treated with ICI therapy at a large tertiary care referral center in northern Thailand. We found that cirAEs are common immune-related toxicities among ICI recipients (estimated incidence occurred in approximately one-third) and mainly include isolated pruritus, eczematous/dermatitis, and skin xerosis. However, there was no significant difference in the incidence of cirAEs according to sex, type of cancer, or ICI therapy. We also found that individuals aged >75 years and with a history of CKD stage 3-4 are at a higher risk of cirAE-related ICI therapy. Based on a clinical perspective, our findings can help inform decision-making management and provide insights for the routine surveillance of cancer patients receiving ICI therapy.

To our knowledge, several mechanisms responsible for cirAE-related ICI therapy, particularly immunopathogenesis *via* the generation of autoreactive T and B cells, have been proposed (3, 6). The estimated incidence of cirAE-related ICI treatment in this study was comparable to the previous literature in melanoma and non-melanoma patients, as well as in Asian and non-Asian patients (5, 7–12, 17). The overall incidence of cirAE-related ICI therapy among cancer patients was 13.1% to 52.3% (this study estimated incidence at 32.1% [95% CI, 24.1-41.4]) (5, 7–12). However, these disparities across different settings could be attributed to variations in the terminology of cirAEs and different types of ICI therapies, such as the use of anti-CTLA-4 or combination therapy. With a wide and diverse range, the reporting of cirAE-related ICI therapy was based on generic terms and skin manifestations (i.e., skin rash or dermatitis) rather than on clinical pathogenesis, immunohistopathological, and phenotypical characteristics. However, for clinical practicability, histopathological examination is considered only in cases with atypical or persistent forms and severe clinical presentation. Taken together, our findings are in line with existing studies that an extensive range of cirAEs could be observed among patients who received ICI therapy, suggesting that our estimated incidence has general relevance (5, 7–12, 17).

Although several cancer-specific risk factors (**Supplementary Table S1**) were investigated, we did not find an association between individual ICI therapies or cancer types and the risk of cirAEs. With respect to a population-based study in the United States, Wongvibulsin et al. (5) suggested that individuals with melanoma (odds ratio [OR], 2.47; 95% CI, 2.11-2.89; *P*<0.001) or renal cell carcinoma (OR, 1.65; 95% CI, 1.36-2.00; *P*<0.001) and use of combination ICI therapy (OR, 1.53; 95% CI, 1.25-1.88; *P*<0.001) had a significantly higher risk of cirAEs compared to other cancer types (lung cancer) or other ICI therapy (pembrolizumab), respectively. Apart from non-white populations, the main reason for the lack of this relationship could be attributable to the study power of analysis, different cancer types, and practice patterns of ICI utilization in our practice. Based on general patient characteristics (**Supplementary Table S1**), we did not observe any association of comorbid conditions such as diabetes, coronary artery disease, and chronic pulmonary disease with subsequent cirAE-related ICI therapy. These findings are supported by several studies that revealed no substantially pre-existing comorbidities and the risk of both cirAEs and non-cirAEs (5, 18, 19). Unfortunately, there is currently no identified specific biomarker for cirAEs; however, if novel biomarkers that predict cirAEs-related ICI therapy are available and established, they could be meaningful in clinical practice.

Unlike a previous study, based on common risk factors, we found that older age (>75 years) and pre-existing CKD stage 3-4 were identified as prognostic risk factors for developing of cirAEs following ICI therapy, which has not been fully elucidated previously. Besides a high rate of multimorbidity, elderly cancer patients also have remodeling of the immune system (20–22). Concerning multiple factors, we postulated that elderly patients could be at risk of cirAEs, in part reflecting the functional changes in immunosenescence and inflammaging. Notably, pre-existing CKD stage 3-4 was also recognized as a predictor of developing cirAE-related ICI therapy in our study (approximately 3-fold compared to those with an estimated glomerular filtration rate >60 mL/min/1.73m^2^). In recent years, Kartolo et al. (23) found that CKD stage 3 or greater was associated with an increased risk of any immune-related adverse events (OR, 10.66; 95% CI, 2.41-47.12; *P*=0.025) among 78 patients receiving ICI therapy. Collectively, our findings may expand and support a previous study demonstrating that poor kidney function may increase the risk of cirAEs *via* the major immune systems, both innate and adaptive responses (24).

## Study strengths and limitations

Our study was based on patient-level data through the retrieval and linking of routinely collected data, providing detailed information on oncology and dermatology practices. Moreover, this study delivers previously under-recognized data on the prevalence and risk factors of cirAEs among Thai cancer patients treated with ICI therapy for cancer types other than melanoma and lung cancer.

Nevertheless, our findings should be exercised in the context of study limitations. First, this study was conducted within a single center; therapeutic strategies and treatment protocols, reimbursement schemes, and access to therapeutic options may differ from other settings, which may limit the generalizability of our findings. Second, given the ambispective design, some information regarding cirAEs was retrospectively collected in some patients; as such, information bias remains. Moreover, an inherent heterogeneous terminology of cirAEs may be present, particularly on the basis of retrospective chart reviews. Generally, reporting cirAEs among patients treated with ICI therapy does not involve dermatologists. However, to address the causality related to ICI therapy, all incidences of cirAEs were based on the ascertainment of a board-certified dermatologist. Third, based on a relatively small number of patients and residual risk factors, associations for the identified risk factors should be interpreted as exploratory. Therefore, validation of our risk findings, including comprehensive sets of candidate risk factors, is necessary. Moreover, subgroup analyses regarding the individual use of ICI therapy and cancer types cannot be performed because of the limited sample size. Fourth, evaluating the use of ICI therapy and severe or potentially life-threatening cirAEs (i.e., severe cutaneous adverse reactions) was not possible because the rates of these events were rare in our setting. Finally, although this study represents real-world evidence in clinical practice, the chronicity of observations and dynamic risk prediction for cirAEs cannot be established over time. Under these circumstances, these findings should not supersede the clinical context and judgment.

## Implications for practice and future research

Despite the study limitations, our findings expand the incidence of cirAEs among broad cancer types other than the white population and suggest the need for routine proactive surveillance of the risk factors. According to the European Academy of Dermatology and Venereology Task Force for Dermatology for Cancer Patients (25), early identification and promptly appropriate treatment strategies are essential to minimize treatment discontinuation and improve patient health outcomes and oncologic treatment. Ultimately, collaborative management-based approaches across oncology and dermatology care practice should be targeted based on comprehensive system assessment concepts to identify cancer patients at high risk of developing cirAEs during ICI therapy.

To date, the development of ICI therapy has emerged as a treatment option in the management of people living with malignancy, resulting in an increasing number of patients receiving ICI therapy; thus, the occurrence of cirAEs is expected to increase. Interestingly, contemporary evidence also suggests that cirAEs may improve the survival of patients treated with ICI therapy (17, 26, 27). Pragmatic trials, along with large proactive pharmacoepidemiology surveillance studies are warranted to fully understand the risk of cirAEs among patients treated with ICI therapy. We underscore the need for a harmonized definition for cirAEs after ICI therapy, which will help inform international pharmacovigilance comparisons and guidelines for managing cirAEs. Moreover, further studies should address the impact of cirAEs on patients’ quality of life in both clinical trials and daily practice to account for the cirAE burden among patients treated with ICI therapy.

In conclusion, we found a relatively common incidence of cirAEs among Thai cancer patients who received ICI therapy. Early identification, particularly in elderly patients and those with CKD, may help optimize therapeutic decision-making, personalized follow-up strategies, collaborative management, and improve health-related quality of life in patients treated with ICI therapy.

## Data availability statement

The raw data supporting the conclusions of this article will be made available by the authors, without undue reservation.

## Ethics statement

The studies involving human participants were reviewed and approved by the Institutional Review Board of the Faculty of Medicine, Chiang Mai University (study code: MED-2562-06587). The patients/participants provided their written informed consent to participate in this study.

## Author contributions

Conceptual design: AL, MC. Data collection: AL, SK, MC, TK, CC, TS, BC, NT, SC, BC. Manuscript writing: AL, SN, SK, MC. Manuscript revision: AL, SK, SN, MC, TK, CC, TS, BC, NT, SC. Study supervision: SN, MC. All authors contributed to the article and approved the submitted version.

## Funding

This work was supported by a Faculty of Medicine Research Fund grant, Chiang Mai University (no.87/2563). This work was also partially supported by a grant from the Pharmacoepidemiology and Statistics Research Center (PESRC) through the Chiang Mai University (ORA2564/635).

## Conflict of interest

The authors declare that the research was conducted in the absence of any commercial or financial relationships that could be construed as a potential conflict of interest.

## Publisher’s note

All claims expressed in this article are solely those of the authors and do not necessarily represent those of their affiliated organizations, or those of the publisher, the editors and the reviewers. Any product that may be evaluated in this article, or claim that may be made by its manufacturer, is not guaranteed or endorsed by the publisher.

## Abbreviations

CI, confidence interval; cirAEs, cutaneous immune-related adverse events; ICI, immune checkpoint inhibitor.
