# Supplementary material for: Incidence and factors associated with cutaneous immune-related adverse events to immune check point inhibitors: An ambispective cohort study
Source: Front Immunol. 2022 Oct 20;13:965550. doi: 10.3389/fimmu.2022.965550 (PMC9630333; doi:10.3389/fimmu.2022.965550)

Supplementary Information

Incidence and Factors Associated with Cutaneous Immune-Related Adverse Events to Immune Check Point Inhibitors: An Ambispective Cohort Study

Athitaya Luangnara, Salin Kiratikanon, Thanika Ketpueak, Thatthan Suksombooncharoen, Chaiyut Charoentum, Busyamas Chewaskulyong, Napatra Tovanabutra, Siri Chiewchanvit, Surapon Nochaiwong*, Mati Chuamanochan*

***Correspondence:**
Mati Chuamanochan, MD, Division of Dermatology, Department of Internal Medicine, Faculty of Medicine, Chiang Mai University, Chiang Mai 50200, Thailand, Phone: +66-53936735-6, Fax: +66-53946234, Email: mati.c@cmu.ac.th

or

Surapon Nochaiwong, PharmD, Department of Pharmaceutical Care, Faculty of Pharmacy, Chiang Mai University, Chiang Mai 50200, Thailand, Phone: +66-899973365, Fax: 6653222741, Email: surapon.nochaiwong@gmail.com

Table S1 Univariable risk factors of cirAE in patients treated with ICI

Factors	Crude RR (95% CI)	*P* value
Age, year		
<65	1.00 (Reference)	
65-75	1.46 (0.78 – 2.72)	0.231
>75	2.04 (1.04 – 2.98	0.038
Sex		
Male	1.00 (Reference)	
Female	0.95 (0.54 – 1.65)	0.849
Canter type		
Lung	1.00 (Reference)	
Liver	1.52 (0.79 – 2.91)	0.209
Melanoma	1.35 (0.56 – 3.26)	0.509
Other†	1.39 (0.65 – 2.96)	0.393
Cancer stage‡		
II/III	1.00 (Reference)	
IV	0.88 (0.49 – 1.57)	0.667
ECOG performance scale‡		
0	1.00 (Reference)	
1	1.61 (0.65 – 3.97)	0.304
2	0.33 (0.04 – 2.61)	0.292
Pre-ICI treatments‡		
Traditional chemotherapy	1.00 (Reference)	
Radiotherapy	0.75 (0.12 – 4.58)	0.752
Targeted agent	1.46 (0.75 – 2.86)	0.269
Concurrent chemoradiation therapy	1.49 (0.47 – 4.78)	0.499
Traditional chemotherapy plus radiotherapy	0.62 (0.10 – 3.95)	0.615
None	1.87 (0.97 – 3.60)	0.063
ICI precipitating first cirAE		
Anti-PD-1	1.00 (Reference)	
Anti-PD-L1	0.89 (0.50 – 1.58)	0.682
Anti-CTLA-4 or anti-CTLA-4 plus anti-PD-1/PD-L1	1.20 (0.39 – 3.73)	0.753
Hypertension		
No	1.00 (Reference)	
Yes	1.50 (0.88 – 2.55)	0.137
Diabetes mellitus		
No	1.00 (Reference)	
Yes	1.74 (0.99 – 3.06)	0.054
Coronary artery disease		
No	1.00 (Reference)	
Yes	2.14 (0.91 – 5.01)	0.080

†Other cancer types included head and neck, genitourinary (renal, urothelial), bladder cancer, colorectal carcinoma, cervical cancer, and gastric cancer.
‡Missing data for one patient.

Abbreviations: CI, confidence interval; cirAEs, cutaneous immune-related adverse events; ICI, immune checkpoint inhibitor; RR, risk ratio.

Table S1 Univariable risk factors of cirAE in patients treated with ICI (continued)

**Factors**	**Crude RR (95% CI)**	***P* value**
Chronic kidney disease: stage 3-4		
No	1.00 (Reference)	
Yes	3.38 (2.52 – 4.52)	<0.001
Chronic pulmonary disease		
No	1.00 (Reference)	
Yes	0.43 (0.07 – 2.71)	0.368
Cirrhosis		
No	1.00 (Reference)	
Yes	0.95 (0.40 – 2.27)	0.911
Charlson comorbidity index		
≤8	1.00 (Reference)	
>8	1.33 (0.78 – 2.28)	0.295
Multimorbidity (excluded cancer)		
No	1.00 (Reference)	
Yes	1.49 (0.84 – 2.63)	0.171
History of drug allergy		
No	1.00 (Reference)	
Yes	0.87 (0.41 – 1.82)	0.705
Hemoglobin, g/dL		
≥12 in females or ≥13 inmales	1.00 (Reference)	
<12 in females or <13 in males	0.99 (0.56 – 1.75)	0.963
White blood cells count x 10^3, µ/L		
≤8.5	1.00 (Reference)	
>8.5	0.90 (0.49 – 1.65)	0.727
Platelet count x 10^3, µ/L		
≤250	1.00 (Reference)	
>250	0.77 (0.45 – 1.32)	0.350
Neutrophil-to-lymphocyte ratio		
≤3	1.00 (Reference)	
>3 – 6	0.80 (0.45 – 1.43)	0.450
>6	0.18 (0.03 – 1.24)	0.082
Platelet-to-lymphocyte ratio		
≤150	1.00 (Reference)	
>150 – 250	0.78 (0.41 – 1.47)	0.440
>250	0.92 (0.46 – 1.83)	0.815
Serum albumin, g/dL		
≥4.5	1.00 (Reference)	
3.5 – < 4.5	0.89 (0.41 – 1.92)	0.771
<3.5	0.69 (0.28 – 1.73)	0.431
Total protein, g/dL		
<8	1.00 (Reference)	
≥8	1.51 (0.88 – 2.58)	0.135

Abbreviations: CI, confidence interval; cirAEs, cutaneous immune-related adverse events; ICI, immune checkpoint inhibitor; RR, risk ratio.

Table S1 Univariable risk factors of cirAE in patients treated with ICI (continued)

**Factors**	**Crude RR (95% CI)**	***P* value**
Globulin, g/dL		
≤3.5	1.00 (Reference)	
>3.5	1.17 (0.67 – 2.02)	0.582
Albumin-to-globulin ratio		
≥1.26	1.00 (Reference)	
<1.26	1.41 (0.70 – 2.87)	0.337
Aspartate aminotransferase, U/L		
≤30	1.00 (Reference)	
>30	1.51 (0.88 – 2.58)	0.135
Alanine transaminase, U/L		
≤30	1.00 (Reference)	
>30	1.38 (0.80 – 2.37)	0.248
Alkaline phosphatase, U/L		
<120	1.00 (Reference)	
≥120	0.98 (0.54 – 1.79)	0.948
Data collection		
Prospective cohort	1.00 (Reference)	
Retrospective cohort	0.54 (0.31 – 0.93)	0.026

Abbreviations: CI, confidence interval; cirAEs, cutaneous immune-related adverse events; ICI, immune checkpoint inhibitor; RR, risk ratio.

Figure S1 Study Flow on the Selection of Eligible Patients

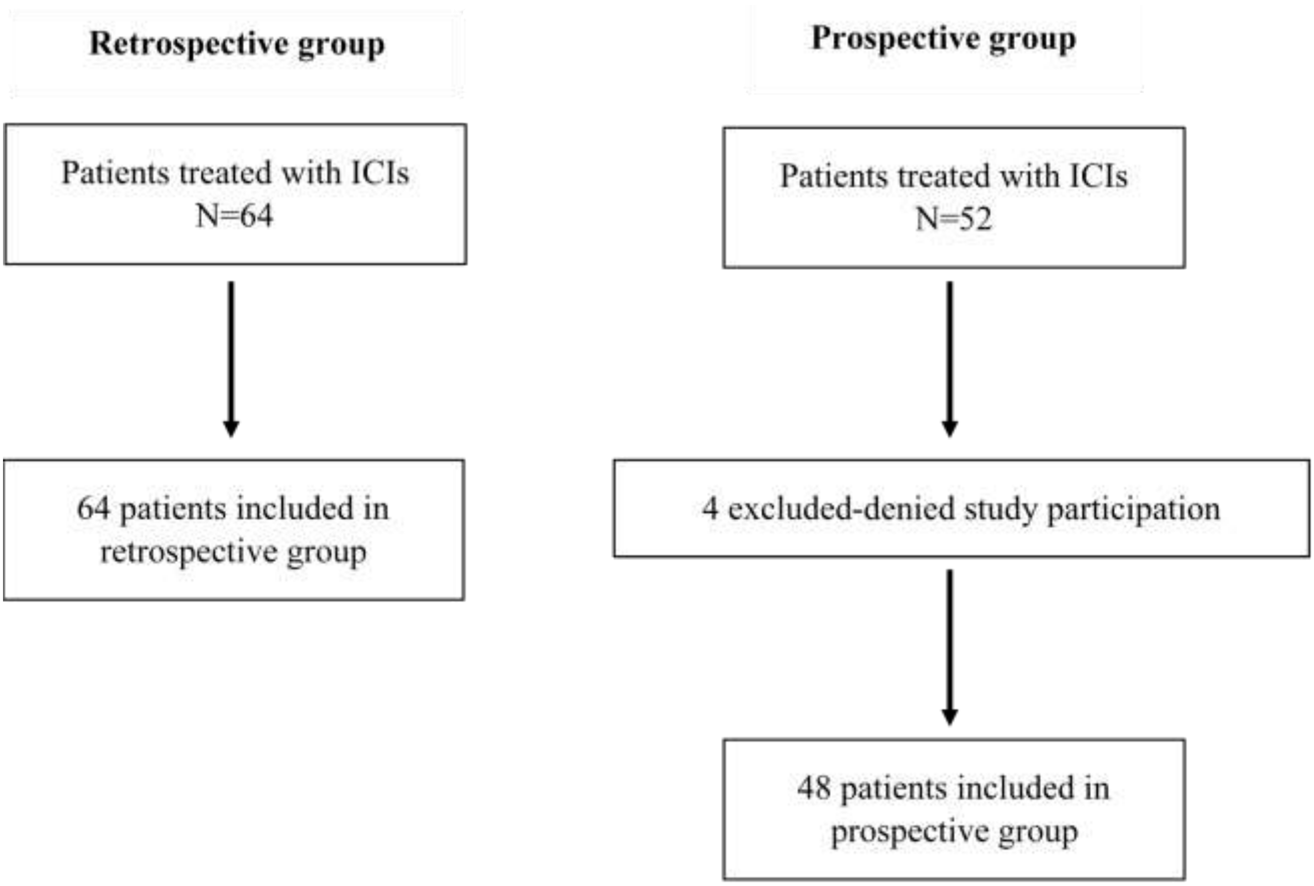

Supplement: Supplementary file 1 [file DataSheet_1.pdf]
